# Dr. Thomas Hun

**Published:** 1896-08

**Authors:** 


### Obituary.

Dr. Thomas Hun, of Albany, died at his residence in that city June 23, 1896. Dr. Hun had been engaged in medical practice for fifty-seven years and was almost the last representative of the old régime. By this we do not mean that he was antiquated in his methods of medical practice, but that in his courtliness of manner, sweetness of disposition and temper, exalted character and scholarly bearing, he represented an element in the medical profession that, sadly enough, seems rapidly fading away.

The Medical Society of the County of Albany, the faculty of Albany Medical College, the medical staff of St. Peter's, Albany, and the Child's hospitals, the board of trustees of Corning Foundation for Christian Work in the Diocese of Albany, the dean and chapter of the Cathedral of All Saints, the trustees of Albany Academy and the regents of the University all have passed appropriate resolutions or memorials, that for beauty of diction and simplicity of style we have rarely seen equaled. We wish we could reproduce them all here, but that is impossible. They may be found in the *Albany Medical Annals* for July, 1896, supplemented by a brief but fitting editorial notice. They all constitute a most lovely tribute to the character and memory of a lovable man. *Requiescat in pace.*

